# The Uptake of Preregistration, and Its Impact on the Proportion of Supported Hypotheses, in Sports Science

**DOI:** 10.1007/s40279-026-02451-2

**Published:** 2026-05-08

**Authors:** Hunter Bennett, Henry Blake, Noah d’Unienville, James Murray, Jordan Fox

**Affiliations:** 1 School of Allied Health and Human Performance, Adelaide University, GPO Box 2471, Adelaide, SA 5001 Australia; 2 Alliance for Research in Exercise, Nutrition, and Activity (ARENA), Adelaide University, Adelaide, Australia; 3 Human Performance Research Centre, School of Sport, Exercise and Rehabilitation, Faculty of Health, University of Technology Sydney, Sydney, Australia

## Abstract

**Background:**

The proportion of studies with results supporting their hypotheses has been implausibly high in sports science, suggesting the widespread presence of questionable research practices (QRPs). Preregistration is recommended to mitigate QRPs.

**Objectives:**

The aim of this study was to evaluate the uptake of preregistration in sports science journals, and its association with the proportion of supported hypotheses. The influence of journal quartile on these factors was also examined. Differences in hypothesis reporting between published manuscripts and original preregistrations were also explored.

**Methods:**

A total of 2006 original research articles published from 19 journals in 2024 were included, of which 201 (10.0%) were preregistered, and 1205 provided a hypothesis (60.0%). Logistic regression analyses assessed whether preregistration frequency and the proportion of supported hypotheses varied across journal quartiles (i.e., quartile 1, 2, 3, and 4, as ranked by SCImago).

**Results:**

The proportion of preregistered studies that had results supporting their hypotheses (45%) were significantly lower than non-preregistered studies (56%) (*X*^2^ (1) 5. 5978; *P* = 0.018), although the effect size was negligible (*V* = 0.07, 95% confidence interval [CI] = 0.01, 0.13). Quartile 3 and 4 journals had less preregistrations (odds ratio [OR] = 0.27, 95% CI = 0.17, 0.41) and supported hypotheses (OR = 0.73, 95% CI = 0.55, 0.97) than quartile 1 journals. Of 134 preregistered papers with a hypothesis, 93 (69.4%) either did not have a hypothesis in their original preregistration or presented a different hypothesis from what was reported in the preregistration.

**Conclusions:**

The uptake of preregistration in sports science is low, and its effectiveness is limited, possibly owing to researchers not adhering to preregistered research plans and adopting QRPs.

**Preregistration:**

This study was prospectively preregistered on the Open Science Framework https://osf.io/c8jfm/files/osfstorage).

**Supplementary Information:**

The online version contains supplementary material available at 10.1007/s40279-026-02451-2.

## Key Points

**Table:** 

Approximately 10% of sports science studies are preregistered.
Preregistered studies report supporting their hypotheses slightly less often than non-registered studies, but the difference is not practically meaningful.
Nearly 70% of preregistered studies report hypotheses that differ from their original preregistration.

## Introduction

There is evidence suggesting researchers both knowingly and unknowingly adopt “questionable research practices” (QRPs) when designing, conducting, analyzing, and reporting research that can bias study results. Some of these practices include [1]: P-hacking, whereby researchers selectively analyze data or conduct multiple statistical tests until a significant result is found, often by adjusting variables or sample inclusion criteria; hypothesizing after the results are known (HARKing), whereby researchers formulate hypotheses on the basis of collected (and observed) data rather than testing prespecified hypotheses; and selective reporting, whereby researchers choose to only publish significant results while omitting null findings. The adoption of QRPs is likely to lead to an increase in the number of type I errors (i.e., false positives) reported in literature, contributing to publication bias and an inability to replicate prior research [2]. This has significant implications for the broader scientific community. Concerns about the replicability of previous research findings may erode public confidence and trust in science [3, 4]. Some have argued that this erosion of trust could fuel antiscience movements [5] and lead to a reduction in research funding [6]. An inability to replicate published research has been demonstrated in the field of psychology [7–10], cancer [11], economics [12], and philosophy [13].

In sports science-related topics, preliminary research has indicated that more than 80% of papers report hypotheses that are supported by study results [14]. This is noteworthy, as even if researchers did hypothesize with a “true” accuracy of 100% (which is very unlikely), for studies aiming to detect a significant effect, a minimum of 20% of those correctly hypothesized studies would be expected to produce nonsignificant results due to chance (false negatives; type II errors) when studies are powered at 80%, a commonly applied standard. This consideration is before factoring in many of the logistical constraints that are common in sports science-related research, such as low sample sizes [15], smaller than predicted effect sizes [16], and high measurement variability [17]—all of which would cause a further increase in the type II error rate. Similarly, a recent examination of almost 1700 articles in sports science-related journals reported a large excess of research with statistically significant results, with *P*-values of just below 0.05, a pattern not expected to occur if the published results were less biased [18]. Collectively, this may imply that sports science research is plagued with QRPs, highlighting the need for active prevention.

Preregistration has emerged as one possible method of mitigating QRPs [19]. Preregistration is the process of prospectively registering a research plan or study protocol prior to that study commencing. These outline the study’s hypotheses, methods, and analysis plans before data collection begins, increasing transparency and accountability in the research process. While there has been a widespread push for researchers to adopt open science practices [20], including preregistration [21], both the frequency and effectiveness of their uptake in sports science is unclear.

The adoption of preregistration varies considerably across research fields. For instance, recent non-peer-reviewed work reported that 43% of studies published in four quartile 1 (Q1) psychology journals had been preregistered [22]. Similarly, analyses of randomized controlled trials published in 2021 across 15 economics journals found a preregistration rate of 40% [23]. In contrast, an investigation of 156 epidemiological studies using data from the Norwegian Mother, Father, and Child Cohort (MoBa) found that only 1.3% had been preregistered [24]. To the authors’ knowledge, only one study has examined the prevalence of preregistered studies in sports science. That study, examining a small sample (*n* = 243) of articles published in Q1 journals, found that approximately 12% were preregistered [25]. However, it is also likely that preregistration differs between journal quartiles. Journal quartiles are rankings that divide journals within a specific subject category into four groups (Q1–Q4) on the basis of their impact or citation performance, whereby Q1 journals represent the top 25% of journals within that category, while Q4 journals represent the lowest 25%. For example, evidence from biomedical research suggests that randomized controlled trials (RCTs) are more likely to be published in higher-quartile journals [26]. Moreover, RCTs are also more likely to be preregistered [27], which suggests that the findings of the previous study may not accurately reflect preregistration practices across the broader field of sports science, including within lower-quartile journals, where RCTs are less common. Furthermore, the study did not investigate whether preregistration was associated with the rate of supported hypotheses, which would have given insight into their effectiveness in sports science.

Given the abovementioned research gaps, this study has three distinct aims: (1) to report on the proportion of original research studies that are preregistered in sports science journals, (2) to determine whether the proportion of supported hypotheses in preregistered studies will be significantly less than those that are not preregistered, and (3) to explore whether the frequency of preregistration and the proportion of supported hypotheses varies across different quartiles (i.e., Q1, Q2, Q3, and Q4, as identified via SCImago) of sports science journals. On the basis of the results of a recent systematic review reporting on the open science practices of Sport Medicine Research in a small (*n* = 243) sample of studies published in 2022, which indicated 12% of papers were preregistered [25], and assuming that the rate of preregistration has increased in sports science in line with other academic disciplines [23, 28], we hypothesize that 15–25% of included articles were preregistered. We also hypothesize that the proportion of supported hypotheses in preregistered studies will be significantly less than those that are not preregistered. Finally, we also hypothesize that higher-quartile journals will have a higher frequency of preregistered papers, and as such, a lower proportion of supported hypotheses, compared with lower-quartile journals.

## Methods

This study employed a cross-sectional design. Original research articles published between 1 January and 31 December 2024 in journals in the SCImago category “Sport Sciences” were eligible for inclusion. Articles were selected from journals across all four quartiles, aiming for a similar number of articles from each quartile. SCImago calculates journal quartiles on the basis of the SCImago Journal Rank (SJR) indicator, which reflects both the number of citations received by a journal and the prestige of the journals where those citations come from. Within each subject category, journals are ranked by SJR score and then divided into four quartiles. Once the journals were identified, Altmetric Explorer was used to identify all articles published from those journals within the specified date range, and to extract their titles, author lists, and DOIs. Journals were chosen at random. However, to account for variations in publication numbers (expecting higher-quartile journals to publish more articles), in the first round of data extraction, articles were extracted from three Q1 journals, four Q2 journals, five Q3 journals, and five Q4 journals. To balance the numbers between quartiles, additional articles were then extracted from one Q1 journal and one Q3 journal (total = 19). Any non-original research articles (i.e., reviews, editorials, consensus statements, letters to the editors, etc.) were excluded, as were articles that implemented a Delphi or qualitative design, as these were considered less likely to have a formal hypothesis.

### Preregistration

This study was prospectively preregistered on the Open Science Framework (https://osf.io/x7eju/overview).

### Extracted Outcomes

The author guidelines of all included journals were examined to describe what recommendations they provide around the inclusion of a hypothesis, and requirements for preregistration (Supplementary Digital Content 1), and to support the forthcoming discussion. Data pertaining to preregistration status, hypothesis testing, and hypothesis testing results were also extracted (Table 1). Finally, the preregistrations of included studies were examined to assess whether the stated hypotheses were consistent with those in the published article.

**Table 1:** Extracted data from included studies

Was the study preregistered (identified via a statement within the manuscript)?	Yes/no
Did the study have a formal hypothesis (defined as a statement in which the article specifically predicted a testable outcome, rather than posing a general research question or aim) provided in the Introduction or Methods?	Yes/no
*If no formal hypothesis was provided*	
Did the study state its analysis was exploratory, or did it clearly state no formal hypothesis was provided?	Yes/no
*If formal hypothesis was provided*	
Did the results of the study align with the hypothesis (yes/no/partially [partially being considered where multiple hypotheses are presented without a clear “primary” hypothesis, and some are supported, and some are not]/unclear [results are not provided for the hypothesized outcome])?	Yes/no/partially/unclear

### Study Procedures

Altmetric Explorer was used to identify all relevant data for articles published within the eligible timeframe from the selected journals, which were then downloaded to a custom Microsoft Excel spreadsheet for eligibility assessment and data extraction. All eligible articles were independently examined by the lead author to extract all outcome measures (*n* = 2006). Once initial data extraction was complete, all included articles were cross-checked by one other member of the research team to evaluate extraction accuracy (JF = 670; JM = 683; ND = 347; HB = 306). Any discrepancies were resolved by consensus among the authorship team. Of the 6018 data items extracted (3 items per study), 176 discrepancies were identified (97.1% agreement), all of which were resolved through consensus. Finally, for each article that reported at least one hypothesis, the total number of hypotheses within the paper and the number that were supported were recorded and reported descriptively.

### Statistical Analysis

To test the first hypothesis, the following data were reported descriptively: the number of total studies examined (across the whole cohort and each quartile), and the proportion that were preregistered and not preregistered (across the whole cohort and each quartile). Then, a one-sample binomial test comparing the observed proportion of preregistered studies with the lower bound of the hypothesized range (15%) was conducted to evaluate whether the observed rate differed significantly from the expected proportion. To test the second hypothesis, the proportion of supported hypotheses between preregistered and non-preregistered studies were compared using a chi-squared test of independence. Cramér’s *V* effect size was also reported to provide insight into the extent to which any differences are practically meaningful, and interpreted using the following thresholds: < 0.1 = negligible, < 0.2 = weak, < 0.4 moderate, < 0.6 = relatively strong, < 0.8 = strong, and > 0.80 very strong [29]. To test the third hypothesis, a logistic regression was conducted to assess whether preregistration frequency and the proportion of supported hypotheses varied across journal quartiles. For preregistration, the dependent variable was preregistration status (yes/no), with journal quartile (Q1, Q2, Q3/Q4) as the independent variable. For hypothesis support, the dependent variable was supported hypothesis (yes/no), with journal quartile as the independent variable. For this analysis, effect sizes were quantified using odds ratios (OR) and considered trivial (0.77–1.00 or 1.00–1.29), small (0.51–0.78 or 1.30–1.99), moderate (0.25–0.50 or 2.00–3.99), and large (≤ 0.24 or ≥ 4.00) [30]. All analysis was conducted in R studio (version 4.3.1). All data and code can be found on the Open Science Framework project linked to this study ( https://osf.io/c8jfm/files/osfstorage).

Assuming that a ~ 10% difference in successful hypothesis rates is practically meaningful, and using the ~ 80% positive hypothesis rate presented in prior research [14], we aimed to detect a difference between proportions of 80% and 70% with an alpha of 0.05 and 80% power. The sample size calculation was based on a two-proportion chi-squared test (independent groups) in G*Power (version 3.1.9.7). Expecting a sampling ratio of 1:6 between preregistered and non-preregistered studies (i.e., ~ 20% of studies being preregistered), we determined that a minimum of 142 preregistered and 708 non-preregistered studies would be required (total *n* = 850). We note that the a priori power calculation was based on the chi-squared comparison. As such, the logistic regression analyses may be at greater risk of false negatives.

### Deviations from Planned Protocol

It was initially proposed that for all formal analysis examining differences between quartiles, quartiles 1–4 would be analyzed independently. However, owing to there being much fewer articles published in Q4 journals than initially anticipated, Q3 and Q4 were combined for analysis. For complete transparency, the preregistered analysis was still conducted, and the results are provided in Supplementary Digital Content 2. Furthermore, the decision to conduct the exploratory descriptive analysis was made after preregistration occurred. Additionally, early data extraction identified a lower preregistration rate than hypothesized, and therefore, to ensure the minimum number of preregistered studies was met, a larger total number of studies was included. Lastly, rather than crosschecking all data, it was initially proposed that 10% of articles would be crosschecked to obtain a measure of extraction accuracy. However, this initial check resulted in 96% agreement between authors, which the authors believed could result in categorization errors for a notable number of the 2006 articles identified. These all related to whether a hypothesis was “fully” or “partially” supported, and occurred when a study had multiple outcome measures contribute to a single hypothesis and was on a topic outside the research team’s direct area of expertise. As such, the authors opted to cross-check all data for discrepancies, which were then resolved through consensus agreement to enhance data extraction and categorization accuracy.

## Results

A total of 2544 articles published from 19 journals (Q1 = 4, Q2 = 4, Q3 = 6, Q4 = 5) were identified through Altmetric Explorer. Of these, 538 did not meet the inclusion criteria, leaving 2006 articles for analysis. Of these 2006 articles, 201 (10.0%) were preregistered, and 1206 provided a hypothesis (60.1%), of which 669 (55.5%) were supported by the results of the study. The observed proportion was lower than expected (*P* < 0.001), with a 95% confidence interval of 8.7–11.4%, indicating that the first hypothesis was not supported. Across these 1206 articles, an average of 1.5 (standard deviation [SD] = 0.8; range = 1–9) hypotheses were reported per article, of which an average of 1.0 (SD = 0.8; range = 0–5.5) were supported. Of the 800 articles that did not provide a hypothesis, 54 (6.8%) described their study as exploratory, or stated that no formal hypotheses were provided. Additional demographic information is presented in Table 2.

**Table 2:** Number of studies per quartile

Quartile	Total	Preregistered (% of total)	Hypothesis provided (% of total)	Hypothesis supported (% of hypothesis provided)
Q1	666	92 (13.8%)	408 (61.3%)	255 (62.5%)
Q2	611	79 (12.9%)	427 (69.9%)	211 (49.4%)
Q3	588	26 (4.4%)	319 (54.3%)	173 (54.2%)
Q4	141	4 (2.8%)	52 (36.9%)	30 (57.7%)

Percentages reported as a percentage of total articles within each quartile

Supplementary Digital Content 1 contains journal-specific information extracted from their respective author guidelines regarding requirements around hypothesis testing and preregistration. With respect to hypothesis testing, three journals (*The American Journal of Sports Medicine*; *Journal of Strength & Conditioning Research*; and *Journal of Sport Rehabilitation*) emphasized including a hypothesis (Q1 = 2, Q3 = 1), four journals (*European Journal of Applied Physiology*; *Journal of Biomechanics*; *Human Movement Science*; and *Journal of Human Sport and Exercise*) mention the inclusion of a hypothesis (Q2 = 1, Q3 = 1, Q4 = 2), and 12 journals (*British Journal of Sports Medicine*; *Scandinavian Journal of Medicine & Science in Sports*; *European Journal of Sport Science*; *Journal of Applied Physiology*; *Research Quarterly for Exercise & Sport*; *International Journal of Sports Medicine*; *Kinesiology*; *Sport Sciences for Health*; *International Journal of Athletic Therapy & Training*; *Motor Control*; *Science and Sports*; and *Sports Engineering*) did not mention hypothesis testing (Q1 = 2, Q2 = 3, Q3 = 3, Q4 = 4). Regarding preregistration, no journals required the prospective preregistration of all studies. However, eight journals (*British Journal of Sports Medicine*; *The American Journal of Sports Medicine*; *Scandinavian Journal of Medicine & Science in Sports*; *Journal of Strength & Conditioning Research*; *European Journal of Applied Physiology*; *European Journal of Sport Science*; *Journal of Biomechanics*; and *Sport Sciences for Health*) did require the preregistration of clinical trials if they met the definition put forward by the World Health Organization or the International Committee of Medical Journal Editors (Q1 = 4, Q2 = 2, Q3 = 2) (both of which define a clinical trial as “any research study that prospectively assigns human participants or groups of humans to one or more health-related interventions to evaluate the effects on health outcomes”) and three journals (*Research Quarterly for Exercise & Sport*; *Human Movement Science*; and *Journal of Sport Rehabilitation*) mentioned preregistration albeit provided no recommendations regarding their use (Q2 = 1, Q3 = 2), while the remaining eight journals (*Journal of Applied Physiology*; *International Journal of Sports Medicine*; *Kinesiology*; *Journal of Human Sport and Exercise*; *International Journal of Athletic Therapy & Training*; *Motor Control*; and *Science and Sports*; *Sports Engineering*) did not mention preregistration at all (Q2 = 1, Q3 = 2, Q4 = 5).

Table 3 outlines the number of preregistered and non-preregistered studies that had a hypothesis, and the number that were supported, partially supported, not supported, and unclear.

**Table 3:** Proportion of supported, partially supported, not supported, and unclear support for hypothesis in preregistered and non-preregistered studies

	Preregistered	Non-preregistered
Total studies	201	1805
Studies with hypothesis	134 (66.7%)	1072 (59.4%)
Supported	61 (45.5%)^a^	608 (56.7%)^a^
Partially supported	35 (26.1%)^a^	303 (28.3%)^a^
Not supported	38 (28.4%)^a^	159 (14.8%)^a^
Unclear if supported	0 (0.0%)^a^	2 (0.2%)^a^
Studies without hypothesis	67 (33.3%)	733 (40.6%)
Described as exploratory	8 (11.9%)^b^	46 (6.3%)^b^

^a^Percentages for hypothesis outcomes are calculated as % of studies with hypothesis

^b^Percentages for exploratory studies are calculated as % of studies without hypothesis

**Fig. 1:**
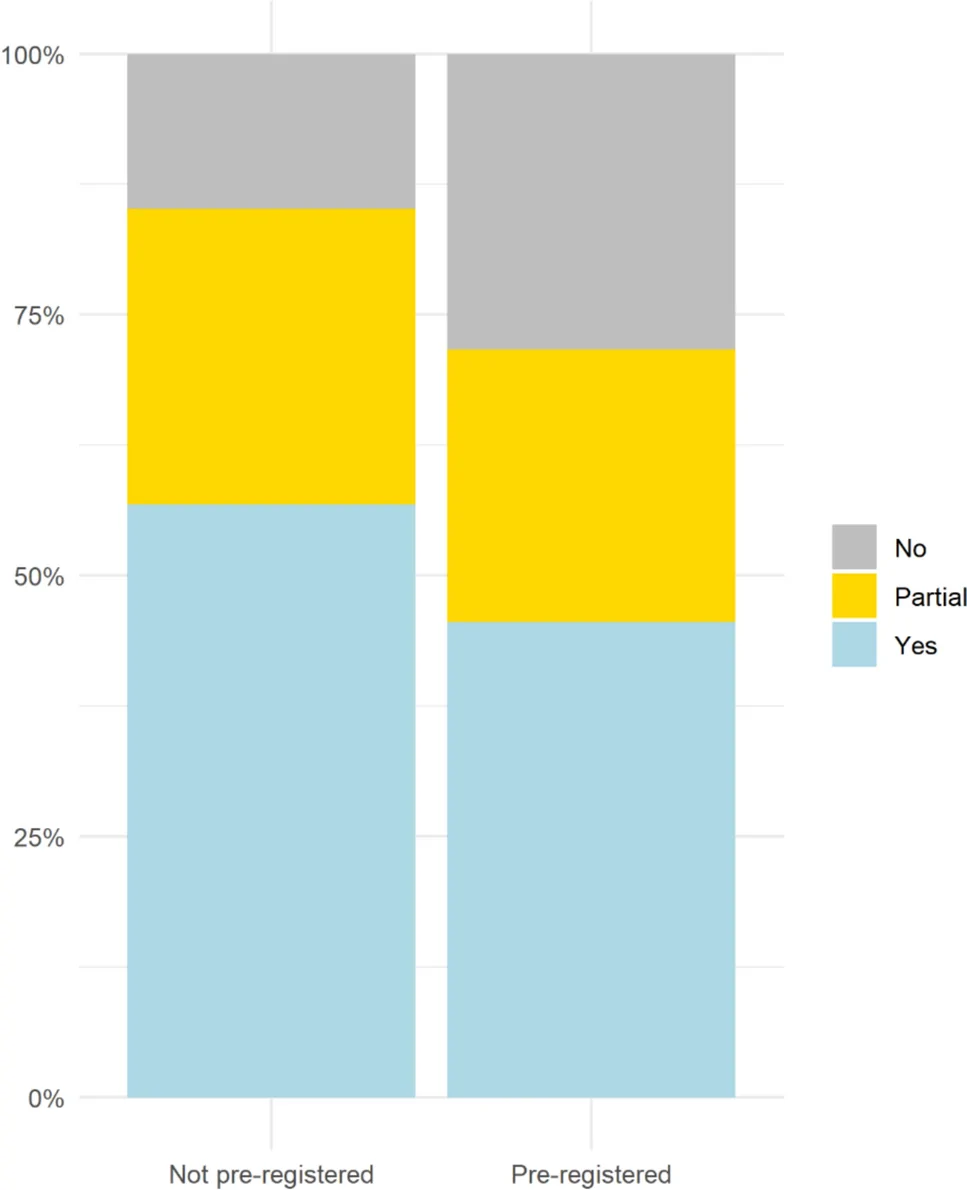
Relative proportion of supported hypotheses among preregistered and non-preregistered studies

The proportion of preregistered studies that had results that fully supported their hypothesis were significantly lower than non-preregistered studies (*X*^2^ (1) = 5.5978; *P* = 0.019), with a negligible effect size (*V* = 0.07, 95% CI = 0.01, 0.13) (Fig. 1), supporting the second hypothesis.

### The Effect of Quartile on the Proportion of Preregistrations and Supported Hypothesis

There were no significant differences in the number of preregistrations between Q1 and Q2 journals, although Q2 journals had a significantly lower rate of supported hypotheses than Q1 journals (small effect), partially supporting the third hypothesis. Conversely, Q3 and Q4, collectively, had a significantly lower number of preregistrations (moderate effect) and supported hypotheses (small effect) than Q1 journals (Table 4), supporting the third hypothesis.

**Table 4:** The differences in preregistration and supported hypotheses proportions between quartiles

Quartile	Preregistration		Supported hypotheses	
	OR (95% CI)	*P*-value	OR (95% CI)	*P*-value
Q1	1.00 (reference)		1.00 (reference)	
Q2	0.93 (0.67, 1.28)	0.643	0.59 (0.45, 0.77)	< 0.001*
Q3/4	0.27 (0.17, 0.41)	< 0.001*	0.73 (0.55, 0.97)	0.031*

*OR* odds ratio, *CI* confidence interval, *Q* quartile

*Indicates significant (*P* < 0.05) difference

### Exploratory Analysis

Of the 134 preregistered articles that provided a hypothesis, a total of 93 (69.4%) either did not have a hypothesis in their original preregistration (*n* = 80, 59.7%) or presented a different hypothesis from what was reported in the original preregistration (*n* = 13, 9.7%). Furthermore, 12 (17.9%) of the 67 preregistered articles that did not report a hypothesis in their published manuscript did provide a hypothesis in their preregistration. Of these 12, 7 (58%) of the non-reported hypotheses were not supported by the results of the study. Finally, one study (0.7%) stated that it was preregistered in the published manuscript but did not provide a registration number or note the trial registry where it was preregistered. As such, for this one article, we were unable to access its preregistration and determine how closely it aligned with what was reported in the published manuscript.

## Discussion

This study provides the first comprehensive evaluation of both the frequency and effectiveness of preregistration in sports science across multiple journals and quartiles. Contrary to our first hypothesis, 10% of all articles were preregistered, which was notably lower than the 15–25% anticipated. Moreover, while the proportion of supported hypotheses in preregistered studies was significantly lower than those that were not preregistered, the effect size was negligible, suggesting the difference may not be meaningful. Regarding our third hypothesis, journals ranked in the top quartile of the Sports Science category (as ranked by SCImago) had a higher proportion of preregistered studies than quartile 3 and 4 journals, although they also had a higher proportion of supported hypotheses. Finally, our exploratory analysis identified a concerning trend whereby more than two-thirds of preregistered studies reported a hypothesis that was not provided in their original preregistration.

### The Uptake of Preregistration in Sports Science

Despite recent calls for greater transparency in sports science research, including recommendations for study preregistration [21, 31], the proportion of preregistered studies in our sample (10%) was slightly lower than previously reported in the literature (~ 12%) [25], even though the previous review examined studies published approximately 2 years earlier. The reason for this discrepancy may be attributed to differences in the samples examined. The earlier review focused exclusively on studies published over a 6-month period in five Q1 sports science journals. In contrast, our sample included journals across all quartiles, and if our results for Q1 journals were isolated, the preregistration rate was 13.8%. Research in biomedicine suggests that randomized controlled trials are more likely to be published in high-impact journals [26], which are in turn more likely to be registered in a clinical trial registry. As such, the inclusion of lower-quartile journals in our analysis likely contributed to the lower overall proportion of preregistered studies. Further supporting this, we found that studies published in lower-quartile journals were significantly less likely to be preregistered than those in higher-quartile journals. Although our finding of a 13.8% preregistration rate in Q1 journals is similar to previous work, it may also reflect a gradual increase in preregistration uptake. However, this trend cannot be confirmed without additional future research.

Interestingly, the lack of uptake in preregistration within sports science contrasts with trends observed in other fields. For instance, the number of preregistered studies in 19 leading experimental economics journals rose from 7 in 2017 to 190 in 2023 [28]. A similar trend was observed in randomized controlled trials published in 15 general economics journals, where the rate of preregistration increased from 14% in 2018 to 40% in 2021 [23]. Additionally, recent non-peer-reviewed work examining four high-quartile psychology journals reported that 43% of studies were preregistered [22], which is notably higher than the current sample. While the reasons for low uptake in sports science remain unclear, potential explanations exist. Although researchers generally view preregistration positively, some perceive they increase overall workload and contribute to greater work-related stress [32]. Others believe it restricts their ability to explore data, but this constraint is arguably a strength as it may help prevent QRPs [33]. Field-specific factors may also play a role. For example, applied sports science studies are often conducted in collaboration with, or led by, practitioners working directly with athletic populations [34]. This can lead to the exploration of routinely collected historical data to “find a result” without a predefined research plan. And while exploratory research plays an important role in the generation of new hypotheses, it is important that it is described as such, otherwise the importance of the findings may be overstated [34]. Furthermore, based on the journals reviewed in this study (Supplementary Digital Content 1), sports science publications rarely require preregistration unless a study meets the strict definition of a clinical trial outlined by the International Committee of Medical Journal Editors [35], which is uncommon in the area. In addition to field-specific factors, cultural aspects are also likely to influence preregistration practices. For instance, psychology has been at the forefront of the open science movement, with widespread adoption of practices such as preregistration and, more recently, registered reports, both of which are notably more common in psychology than in many other disciplines [36, 37]. This may also explain why the present study identified higher preregistration rates than those previously reported in epidemiological research, where secondary analyses of large datasets appear to be less frequently preregistered [24].

### The Impact of Preregistration on Supported Hypotheses

The proportion of studies with a supported hypothesis (~ 55%) was lower than previously reported in sports science, where approximately 73% of hypotheses were fully supported [14]. This discrepancy may be attributable to differences in study samples. The prior study included only 215 articles from high-impact journals, which may have biased the results. Our regression analysis supports this, indicating that studies published in higher-quartile journals were more likely to report supported hypotheses than those in lower-quartile journals. This pattern may reflect a publication bias, whereby trials with novel or positive findings are more appealing to editors and reviewers, and thus more likely to be accepted by high-impact journals, a trend that has been observed in medical research [38].

As initially hypothesized, the proportion of supported hypotheses was significantly lower in preregistered studies (~ 45%) compared with those that were not preregistered (~ 56%). While this may indicate that preregistration can reduce the likelihood of QRPs that result in significant effects, the negligible effect size associated suggests that this may not be having a substantial impact on the field more broadly. Indeed, when considering their low uptake in sports science, it makes sense that their overall effectiveness would remain limited until their use becomes more widespread. To the authors knowledge, three prior studies have explored this question in different subsets of registered and nonregistered studies in the field of psychology, of which two observed a large significant difference [39, 40] and one did not [41]. Although the precise reason for the disparity between the present study and prior research is unknown, there may be some field-related differences at play. Most notably, the replication crisis in psychology has been extensively discussed and well-publicized [6], which may have led to widespread changes (including a greater importance on preregistration and data sharing) that has increased the scientific rigor of the field [42]. Such changes are unlikely to have spread to sports science to the same extent, which may also be reflected by the quality of preregistrations within the current sample.

This finding may also highlight the need for field-specific strategies to mitigate the impact of QRPs. In large-scale epidemiological studies, evidence suggests that when multiple research teams independently analyze the same dataset to answer a common research question, the variability in effect size estimates tends to be small, even when a wide range of statistical approaches are applied [43]. Conversely, similar investigations in the social sciences have shown the opposite pattern, with teams producing widely divergent results and reaching vastly different conclusions despite identical starting conditions [44]. Such discrepancies may partly reflect differing research cultures, particularly the degree of emphasis placed on achieving statistically significant results. For example, in epidemiology, there has been a shift away from traditional null hypothesis significance testing toward the estimation and interpretation of point estimates and their accompanying 95% confidence intervals [45].

### Quality of Preregistrations in Sports Science

Concerningly, our exploratory analysis revealed that more than two-thirds of preregistered articles reported hypotheses that were not included in their original preregistration. This issue has been documented in previous research. For example, an examination of 459 registered studies published in psychology journals found that 52% omitted originally preregistered hypotheses, while 57% added new, unregistered ones [46]. Similar concerns were raised in a subset of 300 preregistered psychology studies, which frequently lacked methodological detail and included undisclosed deviations, both of which may increase the risk of false positives occurring [47].

These findings may suggest that authors publishing in sports science journals may frequently engage in QRPs, such as selective reporting and HARKing, to make their findings more appealing. They also indicate a potential lack of attention to preregistrations during the peer-review process. Supporting this concern, a study of 201 preregistered articles published in the *PLOS* journal family with open peer-review histories found that only 18% of reviewers or editors mentioned the preregistration in their peer review reports, and just 10% discussed the relationship between the preregistration and the final manuscript [48]. Considering these findings, we recommend that journal editors require peer reviewers to explicitly evaluate the alignment between study preregistrations and submitted manuscripts as part of the review process. This also highlights the importance of researchers clearly reporting any deviations from their preregistered protocols in their published manuscripts. Importantly, such deviations are not necessarily indicative of QRPs, and may arise from practical challenges encountered during the research process or even be implemented to strengthen the study design [49]. Moreover, deviations are not always initiated by the authors. For instance, reviewers may recommend the removal of nonsignificant results during the peer-review process.

### Future Recommendations for Sports Science

Despite recommendations for sports science to adopt more transparent research practices, the present study suggests that they are not being widely adopted or appropriately used. As such, we believe the following actions would be advisable to improve research practice, and by extension, help prevent the continued infiltration of QRPs into the published literature:

- The transition to compulsory preregistration: As outlined earlier, when properly applied, preregistration can help prevent QRPs by limiting researchers to preplanned primary analyses. If journals required preregistration as a condition of submission, this could meaningfully improve research quality across the field.
- The widespread adoption of registered reports: While preregistration can reduce QRPs, there remains a risk that null findings are not published (i.e., the file drawer problem), leading to publication bias [50]. Registered reports may mitigate this risk. In a registered report, authors submit a study protocol that details the research questions, hypothesis, methodology, and analysis plan to a scientific journal for review prior to commencing the study, as an original research submission. Then, if the registered report is accepted, the journal agrees to publish the study regardless of the findings provided the protocol is followed [51]. Research in psychology has indicated that registered reports may provide a meaningful way to prevent QRPs and type 1 errors [52], although few sports science journals have adopted this practice [53, 54]. If more journals accepted registered reports, it would incentivize researchers to adopt open science practices by providing a clearer pathway to publication. Additional mechanisms to encourage their uptake could include offering expedited review processes for subsequent studies linked to an approved registered report or integrating registered report submission into grant funding requirements.
- The requirement to review preregistrations: On the basis of the results of the present study and prior findings [48], it appears likely that preregistrations in sports science are not routinely evaluated by journal editors or reviewers. Requiring peer reviewers to assess whether manuscripts align with their preregistered protocols would ensure deviations are acknowledged, increasing transparency and potentially reducing the incidence of type I errors.

### Limitations

These findings should be interpreted considering several limitations. Firstly, owing to an unexpectedly low number of published studies in Q4 journals, we combined Q3 and Q4 journals into a single category. This decision limited our ability to assess differences in the proportion of registered studies and supported hypotheses across quartiles. Secondly, owing to the cross-sectional nature of this research, it cannot be ascertained whether the discrepancies between preregistrations and published manuscripts were the result of QRPs, or the authors feeling pressured to provide a hypothesis to ensure author guidelines were met appropriately. Moreover, while discrepancies between preregistrations and final articles were identified, the nature and extent of these deviations were not systematically described. Similarly, whether the hypotheses were tested using appropriate statistical methods was not considered. Future research should consider conducting a detailed analysis of preregistrations to identify both the most common types of deviations, and the appropriateness of the statistical approach used to answer reported research questions. Thirdly, what was considered to have “partial” support varied between studies. For instance, some studies had multiple hypotheses where only one was supported (and the remaining were not), while others had multiple hypotheses where all but one were supported. Fourth, only quantitative primary research studies were included. It is possible that systematic reviews or qualitative studies in sports science exhibit different preregistration rates than those reported here. Finally, while all extracted data were cross-checked for accuracy, the categorization was likely limited by the research team’s content-specific knowledge. For example, many studies presented multiple primary outcomes but described their hypotheses with minimal detail. In such cases, the research team had to rely on the authors’ interpretation of whether the results aligned with the stated hypotheses, which could have been made to sound more definitive and compelling to enhance the appeal of the paper to the submitted journal. Ideally authors should provide greater clarity on reported hypotheses by offering clear predictions regarding research outcomes, including the direction of relationships between variables and differences between groups [55].

## Conclusions

This study presents the first comprehensive evaluation of both the frequency and effectiveness of preregistration in a large sample of sports science articles across journals spanning all quartiles. Overall, 10% of eligible articles were preregistered. The proportion of supported hypotheses was significantly lower in preregistered studies compared with non-registered studies, although with a negligible effect size observed. Higher-quartile journals published a greater proportion of preregistered studies and reported more supported hypotheses than lower-quartile journals. Notably, more than two-thirds of preregistered studies contained a hypothesis that was not included in their original preregistration. These findings suggest that the uptake of preregistration in sports science remains low, and its effectiveness appears limited. This may be partially attributable to inconsistent adherence to preregistered protocols and the presence of QRPs during manuscript preparation.

## Supplementary Information

Below is the link to the electronic supplementary material.

Supplementary file1 (DOCX 26 KB)

Supplementary file2 (DOCX 14 KB)

## Declarations

### Data availability statement

All data and code can be found on the Open Science Framework project linked to this study (https://osf.io/c8jfm/files/osfstorage).

### Code availability statement

All data and code can be found on the Open Science Framework project linked to this study (https://osf.io/c8jfm/files/osfstorage).
