# Supplementary material for: The Uptake of Preregistration, and Its Impact on the Proportion of Supported Hypotheses, in Sports Science
Source: Sports Med. 2026 May 8;56(9):2283–93. doi: 10.1007/s40279-026-02451-2 (PMC13499854; doi:10.1007/s40279-026-02451-2)
Supplement: Supplementary file 1 [file 40279_2026_2451_MOESM1_ESM.docx]

# Author Guidelines of included journals: Pre-registration and Hypothesis testing.

Table 1: Key for colour codes in table 2.

| **Colour** | **Hypothesis** | **Pre-registration** |
| --- | --- | --- |
|  | Journal emphasizes the inclusion of a hypothesis (without explicitly stating they are required). | Journal requires the pre-registration of clinical trials if they met the definition put forward by the World Health Organisation (WHO) or the International Committee of Medical Journal Editors (ICMJE). |
|  | Journals mentions the inclusion of a hypothesis. | Journals mentions pre-registration (although provides no recommendations regarding their use). |
|  | Journal does not mention hypothesis testing. | Journal does not mention pre-registration. |

Table 2: Pre-registration and Hypothesis testing information, obtained from author guidelines on the 12^th^ of February 2025.

| **Journal** | **Quartile** | **Hypothesis** | **Pre-registration** |
| --- | --- | --- | --- |
| British Journal of Sports Medicine: | 1 | No statements regarding hypothesis testing or the requirement to include a hypothesis. | Statement regarding pre-registration does not include a requirement to pre-register all trials, just that clinically registered trials must have a data sharing plan:  “To adhere to ICMJE guidelines, we require that a data sharing plan must be included with trial registration for clinical trials that begin enrolling participants on or after 1st January 2019. Changes to the plan must be noted in the Data Availability Statement and updated in the registry record.” |
| The American Journal of Sports Medicine | 1 | Doesn’t state you must have a hypothesis but suggests you should have a hypothesis or purpose in the abstract:  “State the theory(ies) that you are attempting to prove or disprove by your study or the purpose if no hypothesis exists.” | States “clinical trials” must be registered:  “All clinical trials started after January 1, 2016 must prospectively registered at ClinicalTrials.gov or a similar database recognized by the ICMJE to be considered for publication. See list of ICMJE acceptable registries at http://www.icmje.org/about-icmje/faqs/clinical-trials-registration/.The original study protocol for all registered clinical trials must be included and can be uploaded as a supplemental file.” |
| Scandinavian Journal of Medicine & Science in Sports | 1 | No statements regarding hypothesis testing or the requirement to include a hypothesis. | States “clinical trials” must be registered:  “Journals that publish clinical trials should make prospective registration in suitable recommended registries (e.g. ClinicalTrials.gov) a requirement for publication of such trials.” |
| Journal of Strength & Conditioning Research | 1 | Doesn’t state you must have a hypothesis, but does refer to it in the guidelines:  Introduction: “This section is a careful development of the hypotheses of the study leading to the clear purpose of the investigation.”  Methods: “'Experimental Approach to the Problem,'' where the author(s) show how their study design will be able to test the hypotheses developed in the introduction and give some basic rationales for the choices made for the independent and dependent variables used in the study”  Discussion: “Discuss the meaning of the results of your study in this section. Relate them to the literature that currently exists and make sure you bring the paper to completion with each of your hypotheses.” | States the following in regard to preregistration:  “Regarding publication of clinical trials, registration of a trial in a public registry is recommended. The Editor does not advocate a particular registry but recommends the registry meets the criteria set out in the statement of policy of the International Committee of Medical Journal Editors (www.icmje.org). An acceptable registry should include the minimum 20-item trial registration dataset.” |
| European Journal of Applied Physiology | 2 | Does not state a hypothesis must be included, although does say abstract must include aim OR hypothesis:  “The sections should describe briefly and concisely the background and aim/hypothesis of the investigation…”  They also make the following comment with respect to the results:  “The text in the results section should be presented with focus on physiology and the outcome of statistical analyses should generally be limited to those used to test specified hypotheses,” – | States “clinical trials” in line with the World Health Organization (WHO) definition must be registered:  “Authors must register prospective clinical trials (phase II to IV trials) in suitable publicly available repositories” and “the trial registration number (TRN) and date of registration should be included as the last line of the manuscript abstract.” |
| European Journal of Sport Science | 2 | No statements regarding hypothesis testing or the requirement to include a hypothesis. | States “clinical trials” must be registered:  “Journals that publish clinical trials should make prospective registration in suitable recommended registries (e.g. ClinicalTrials.gov) a requirement for publication of such trials.” |
| Journal of Applied Physiology | 2 | No statements regarding hypothesis testing or the requirement to include a hypothesis. | No statements regarding pre-registration. |
| Research Quarterly for Exercise & Sport | 2 | No statements regarding hypothesis testing or the requirement to include a hypothesis. | No statements regarding the requirement of pre-registration, but do mention the following under the heading “data sharing policy”:  “At the point of submission, you will be asked if there is a data set associated with the paper. If you reply yes, you will be asked to provide the DOI, pre-registered DOI, hyperlink, or other persistent identifier associated with the data set(s). If you have selected to provide a pre-registered DOI, please be prepared to share the reviewer URL associated with your data deposit, upon request by reviewers.” |
| International Journal of Sports Medicine | 3 | No statements regarding hypothesis testing or the requirement to include a hypothesis. | No statements regarding pre-registration. |
| Journal of Biomechanics | 3 | No statement regarding the requirement of hypothesis inclusion, but do mention the following when discussing the submission of original research articles:  “Original articles typically explore some explicit biological hypothesis or report original but substantial observations or data of broad utility.”  And the following in when discussing the guidelines for reporting statistical procedures:  “When hypothesis-testing procedures are described in the Methods section, authors should also (1) clearly state the hypotheses to be tested, and justify the hypotheses, in the Introduction section; and (2) evaluate the hypotheses based on the results of the study in the Discussion section.” | States “clinical trials” must be registered:  “The Journal of Biomechanics has adopted the proposal from the International Committee of Medical Journal Editors (ICMJE) which require, as a condition of consideration for publication of clinical trials, registration in a public trials registry. Trials must register at or before the onset of patient enrolment. The clinical trial registration number should be included at the end of the abstract of the article. For this purpose, a clinical trial is defined as any research project that prospectively assigns human subjects to intervention or comparison groups to study the cause-and-effect relationship between a medical intervention and a health outcome. Studies designed for other purposes, such as to study pharmacokinetics or major toxicity (e.g. phase I trials) would be exempt.” |
| Kinesiology | 3 | No statements regarding hypothesis testing or the requirement to include a hypothesis. | No statements regarding pre-registration. |
| Human Movement Science | 3 | No statement regarding the requirement of hypothesis inclusion, but do mention the following:  “Human Movement Science will consider hypothesis-driven analyses and exploratory analyses for which the need for exploration can be motivated.” | No statements regarding the requirement of pre-registration, but do mention the following when discussing the journal aims and scope:  “Through pre-registration of hypotheses and methodology and results-independent editorial decisions, this article type neutralizes many questionable research practices incentivised by today's publish-or-perish pressures.” |
| Journal of Sport Rehabilitation | 3 | No statement regarding the requirement of hypothesis inclusion, but do mention it via the following statement regarding the introduction:  “This section must also specifically state the purpose and hypotheses of the study”  And the following when discussing statistical analysis:  “This section should clearly outline the analytic approach used to test the a priori hypotheses.” | No statements regarding the requirement of pre-registration, but do make the following suggestion in the instance where a study is pre-registered:  If the study was a registered clinical trial, indicate “registration number XXX” and include the actual registration number in the Title Page file” |
| Sport Sciences for Health | 3 | No statements regarding hypothesis testing or the requirement to include a hypothesis. | States “clinical trials” in line with the WHO definition (not all trials) must be registered:  “Authors must register prospective clinical trials (phase II to IV trials) in suitable publicly available repositories” and “the trial registration number (TRN) and date of registration should be included as the last line of the manuscript abstract.” |
| Journal of Human Sport and Exercise | 4 | No statements regarding hypothesis testing or the requirement to include a hypothesis, but do make the following comment when discussing analysis:  “Describe the analyses applied to the data. It is helpful if you arrange this section to be coherent with the hypotheses.” | No statements regarding pre-registration. |
| International Journal of Athletic Therapy & Training | 4 | No statements regarding hypothesis testing or inclusion. | No statements regarding pre-registration. |
| Motor Control | 4 | No statements regarding hypothesis testing or inclusion. | No statements regarding pre-registration. |
| Science and Sports | 4 | No statements regarding hypothesis testing or inclusion. | No statements regarding pre-registration. |
| Sports Engineering. | 4 | No statements regarding hypothesis testing or inclusion. | No statements regarding pre-registration. |
