# Supplementary material for: The Uptake of Preregistration, and Its Impact on the Proportion of Supported Hypotheses, in Sports Science
Source: Sports Med. 2026 May 8;56(9):2283–93. doi: 10.1007/s40279-026-02451-2 (PMC13499854; doi:10.1007/s40279-026-02451-2)
Supplement: Supplementary file 2 [file 40279_2026_2451_MOESM2_ESM.docx]

Table 1: The differences in pre-registration and supported hypotheses proportions between quartiles (pre-planned analysis, with Q3 and Q4 studies kept separate)

| Quartile | Pre-registration | | Supported hypotheses | |
| --- | --- | --- | --- | --- |
|  | OR (95%CI) | P value | OR (95%CI) | P value |
| Q1 | 1.00 (reference) |  | 1.00 (reference) |  |
| Q2 | 0.93 (0.67, 1.28) | 0.643 | 0.59 (0.45, 0.77) | <0.001 |
| Q3 | 0.29 (0.18, 0.45) | <0.001 | 0.72 (0.53, 0.96) | 0.027 |
| Q4 | 0.18 (0.06, 0.45) | 0.001 | 0.82 (0.46, 1.49) | 0.516 |
